# Effect of feeding different levels of *Azolla pinnata* on blood biochemicals, hematology and immunocompetence traits of Chabro chicken

**DOI:** 10.14202/vetworld.2015.192-198

**Published:** 2016-02-20

**Authors:** Deepesh Bharat Mishra, Debashis Roy, Vinod Kumar, Amitav Bhattacharyya, Muneendra Kumar, Raju Kushwaha, Shalini Vaswani

**Affiliations:** 1 Department of Animal Nutrition, College of Veterinary Science and Animal Husbandry, U.P. Pandit Deen Dayal Upadhyaya Pashu Chikitsa Vigyan Vishwavidyalaya Evam Go Anusandhan Sansthan, Mathura - 281001, Uttar Pradesh, India; 2 Department of Poultry Science, College of Veterinary Science and Animal Husbandry, U.P. Pandit Deen Dayal Upadhyaya Pashu Chikitsa Vigyan Vishwavidyalaya Evam Go Anusandhan Sansthan, Mathura - 281001, Uttar Pradesh, India

**Keywords:** *Azolla pinnata*, blood biochemicals, hematology, immunocompetence traits

## Abstract

**Aim:**

The present study was conducted to see the effect of feeding different levels of *Azolla* meal on blood biochemicals, hematology and immunocompetence traits of Chabro chicken.

**Materials and Methods:**

The study was conducted on 160 Chabro chicks, which were randomly divided into four treatment groups each with four replicates of 10 birds. The first treatment (T_1_) served as a control in which basal diets was offered without *Azolla* supplementation while in T_2_, T_3_, and T_4_ groups, basal diet was replaced with *Azolla* meal at 5%, 7.5%, and 10% levels, respectively. A feeding trial was conducted upto 8 weeks. At the last week of trial, blood samples were collected randomly from one bird of each replicate and plasma was separated to estimate certain biochemical parameters, some blood metabolites, minerals and enzymes like alanine aminotransferase and aspartate aminotransferase (AST). Hematological parameters such as hemoglobin, packed cell volume, total leukocytes count and differential leukocytes count were estimated in fresh blood just after collection. The humoral immune response was measured against sheep red blood cells,and cell-mediated immune response was measured against phyto hemagglutinin lectin from *Phaseolus vulgaris* (PHA-P).

**Results:**

The study showed that hematological profile of the Chabro bird was not affected by any treatment except heterophil and lymphocyte which was found higher in T_2_ and T_3_ groups and eosinophil was found higher in a T_3_ group than control. Blood glucose, creatinine, cholesterol, total protein, albumin, uric acid, and triglycerides were found similar in all the groups and within the normal values for broiler chicken. Liver enzymes and macro mineral content in blood were found similar in all the treatment groups and within normal physiological range. Although AST was found higher in 10% replacement group than control, the value was within normal range for broiler chicken. Although antibody titer was found similar in all the experimental groups in the present study, cell-mediate immune response (response to PHA-P) was found higher in 5%, 7.5%, and 10% replacement groups than control(p<0.05).

**Conclusion:**

Similar blood biochemical parameters and higher cell-mediated immune response in *Azolla* replacement group indicated immune-modulatory effect of *Azolla* meal without any toxicity.

## Introduction

In recent years, there has been a steep rise in poultry production throughout the world. As a result, it is gradually becoming a major thrust in the world economy, especially in the livestock sector. This increase has resulted in competition with the conventional human food ingredients leading to shortage and increased the cost of conventional feed ingredients [1]. Hence, some serious attempts were made by nutritionists in the past few years to substitute the conventional feed ingredients with animal wastes, slaughterhouse wastes, different non-conventional feed resources, etc.

*Azolla* is a small aquatic fern which flows on the water surface. The name is referred to conjugation of two Greek words, azo (to dry) and allyo (to kill) because the fern is killed by drought. Use of *Azolla* was initially limited as green manure but its use as mosquito inhibitor [2], herbicide, water saver, water purifier, nitrogen fertilizer saver [2], as drug, for reclaiming saline soils [3] and as bioremediation [4,5] are also been investigated. *Azolla* hosts symbiotic blue-green algae, *Anabaena azollae*, which is responsible for the fixation and assimilation of atmospheric nitrogen. *Azolla*, in turn, provides the carbon source and favorable environment for the growth and development of the algae. It is this unique symbiotic relationship that makes *Azolla*, a wonderful plant with high protein content.

Considering its nutrient content [6,7], *Azolla* was started to be used as feed ingredients for poultry, pig and livestock species. Though variable results were observed, most of the researches suggested improvement on production and reproduction parameters in poultry bird when birds were fed with *Azolla* meal replacing basal diets upto a certain level. Backyard poultry farming is now-a-day's promoted in India considering socio-economic condition of Indian farmers. Different central Government agencies are developing several strains of poultry birds for backyard farming. Chabro is one of them. This is developed by Central Poultry Development Organization (CPDO). Considering the potential of using *Azolla* meal as a partial replacement of commercial broiler feed in Chabro bird, current study was designed to observe effect of feeding different levels of *Azolla pinnata* on blood biochemicals, hematology and immunocompetence traits of Chabro chicken.

## Materials and Methods

### Ethical approval

Experiments were carried out in accordance with the guidelines laid down by the institute Animal Ethics Committee for the use of poultry birds.

### Experimental design, housing and management

In the experiment, there were four treatments groups each with four replicates of ten birds. The first treatment (T_1_) served as control in which basal diets was offered without *Azolla* supplementation while in T_2_, T_3_ and T_4_ groups basal diet was replaced with *Azolla* meal at 5%, 7.5%, and 10% levels, respectively. For experimental feeding trial, 160 days old Chabro broiler chicks were procured from poultry farm, U. P. Pandit Deen Dayal Upadhyaya Pashu Chikitsa Vigyan Vishwavidyalaya Evam Go Anusandhan Sansthan (DUVASU), Mathura and reared for 8 weeks. Chicks were kept on deep litter system in brooder house under standard managemental and hygienic conditions for 1 week. They were provided standard broiler starter ration on newspaper spread on the floor. After 1 week, chicks were individually weighed and randomly divided into four groups of 40 chicks each, on the basis of average uniform body weights, discarding the extreme ranges of body weights. The chicks were transferred replicate wise (10 birds in each replicate groups) in the pens of similar dimensions. The chicks were housed in deep litter system. All the housing and manage mental conditions were similar for different treatment groups. Fresh and clean water was provided *ad libitum* to the chicks of different treatment groups. Throughout the experimental period, the chicks were provided artificial light by electric bulbs of sufficient intensity.

### Feeds and feeding schedule

Standard broiler feeds for starter (0-4 weeks) and finisher (4-8 weeks) periods as per BIS specifications [8] were formulated and prepared in Feed Unit at ILFC, DUVASU, Mathura. Feed was prepared for feeding of experimental Chabro birds was consisted of maize, rice police, soybean meal, fish meal, dicalcium phosphate, lime stone, premix, salt, and metheonine in basal diet. The composition of feeds for the starter and finisher periods are presented in Table-1. In the treatment group, calculated amount of *Azolla* replaced basal diets at different levels. *Azolla* was collected from “*Azolla* Demonstration Unit” of the university and then it was dried under the sun. After drying, it was grounded and stored in the plastic bags until used for feeding. Feed was offered daily to the birds at 8.00 A.M. Representative samples of *Azolla pinnata* powder and experimental feed of starter (T_1_, T_2_, T_3_ and T_4_ groups) and finisher (T_1_, T_2_, T_3_, T_4_ groups) was analyzed for their nutrient composition *viz*. dry matter (DM), crude protein (CP), ether extract (EE), total ash, crude fiber (CF), Ca and P as per AOAC [9]. Fiber fractions of feed samples were determined as per the method of Van Soest and Robertson [10].

**Table-1:** Composition of experimental basal diet (g/kg as DM).

Attributes	Experimental basal diet	

	Starter	Finisher
Ingredients (g/kg) as feed		
Maize	515.5	575.5
Rice police	70	100
SBM	300	250
FM	80	40
DCP	10	11
LS	12	12
Premix	05	05
Salt	06	06
Meth	1.5	0.5
Total	1000	1000

1 kg premix contains vitamin A - 10,000,000 IU, vitamin D3 – 2,000,000 IU, vitamin B2 – 4g, vitamin E -1500 unit, vitamin K-2g, Pantothenate -5 g, nicotinamide-20 g, vitamin B 12-12 g, cholinchloride- 300 g, Ca- 1500 g, Mn -55 g, I -2g, Fe -15 g, Zn -30 g, Cu - 4 g, Co- 0.9 g, DCP=Dicalcium phosphate, SBM=Soybean meal, FM=Fish meal, LS=Limestone, DM=Dry matter

### Blood sampling, hematology and biochemical analysis

At the last week of trial, blood samples were collected randomly from one bird of each replicate in heparinized vial. A part of blood sample was used for hematological parameters. Blood samples were centrifuged at 3000 rpm for 10 min for plasma separation. Separated plasma samples were used for biochemical and enzyme analysis. Glucose and enzymes estimation were done within 24 h of the collection while for other estimations plasma was stored in refrigerator until the analysis. Hemoglobin, packed cell volume, total leukocyte count and differential leukocyte count were done by standard laboratory procedure. Blood biochemicals, liver enzymes activities and macro-minerals were estimated by using kit (Span Diagnostics Ltd.).

### Humoral immune response against sheep red blood cells (SRBC)

Blood from jugular vein of healthy sheep was collected in Alsever's solution. The red blood cells were washed thrice in phosphate buffer saline (PBS, pH 7.2). Finally 1% suspension of SRBC in PBS (V/V) was prepared and stored in a refrigerator at 4°C until its use.

### Immunization and harvesting of immune serum

1 ml of 1% (V/V) of SRBC suspension was injected to the birds. At 5 days post immunization about 2 ml of blood was collected from jugular vein. The antibody titer was determined by hemagglutination assay (HA) methods [11]. The titers were expressed as log 2 value. The response titer was the result of the difference between HA titer before and after SRBC immunization.

### 2-Mercaptoethanol resistant and sensitive antibodies (MER and MES) against SRBC

Antibodies were determined by means of a mercapto ethanol (ME) HA test as per the method described by Martin *et al*. [12] and the titer was recorded as 2-ME resistant antibody and expressed as log 2 values. The reduction of total titer due to 2-ME treatment was called 2-ME sensitive antibody, and the titer was expressed as log 2 value (Total HA titer-MER=MES). MER was denoted as immunoglobulin (Ig) G and MES was denoted as IgM.

### *In vivo* cell mediated immune response

The cellular immune response was assessed by cutaneous basophilic hypersensitivity test *in vivo* by using phyto hemagglutinin lectin from *Phaseolus vulgaris* (PHA-P). Birds were injected intra dermally between 3^rd^ and 4^th^ toe of the right foot or on the wattle with 0.1 mg PHA-P in 0.1 ml of PBS (1 mg PHA-P/ml of PBS). The left foot received 0.1 ml of PBS and served as control. The thickness of inter-digital skin or wattle was measured using micrometer (AMES) at 0 and 24 h after injection. The skin swelling was calculated by substracting the skin thickness at 0 h from that of after 24 h of injection. The foot web index (FI) or wattle index was determined as the difference between inter-digital or wattle swelling values of PHA-P injected and control foot or wattle.

FI or Wattle index (in mm) = (thickness after 24 hinjection of PHA-P of right foot or wattle-thickness before injection of the same foot or wattle) - (thickness after 24 h of injection of PBS of left foot or wattle - thickness before 24 h of injection of PBS of the same foot or wattle).

### Statistical analysis

Data obtained were subjected to analysis completely randomized design with the simple analysis of variance technique [13] using Statistical Package for the Social Sciences [14]. Homogenous subsets were separated using Duncan's multiple range test described by Duncan [15]. Differences among treatments were considered to be significant when p≤0.05.

## Results and Discussion

### Chemical composition of experimental diet

The chemical composition of *Azolla* meal and experimental diet is given in Table-2. The proximate principles, i.e., DM, organic matter, total ash, CP, EE, CF and nitrogen free extract (NFE) of *Azolla* meal were found to be 9.88%, 78.33%, 21.67%, 25.64%, 3.15%, 17.29% and 32.25%, respectively. The cell wall constituents, i.e. neutral detergent fiber and acid detergent fiber and Ca and P contents of *Azolla* meal were found to be 51.15%, 43.38% and 11.12%, 5.91%, respectively. The chemical composition of *Azolla* was comparable with the previous observations of different scientists [16-22]. The experimental diet fulfilled all the nutrient requirement of broiler birds according to BIS [8] CF content of *Azolla* replaced diet was found higher than control as *Azolla* contained more fiber. The mineral content and CPincreased gradually with increasing replacement of basal diet with *Azolla* meal. This was due to more mineral and protein content of *Azolla* meal than the basal diet, whereas the EE decreased slightly in *Azolla* replaced diet. Similar to our findings, the CP content of *Azolla* meal was reported in the range of 21.4-25.78% [16-18]. In contrary to this, Ali and Leeson [23] obtained lower CP content of 16.5-17.67% in *Azolla* meal. The 17.29% crude fiber obtained in the present study is slightly higher than the findings of Parthasarathy *et al*. [24] who reported the CF content of *Azolla* in the range of 13.19-16.54%. The EE content of *Azolla* in the present study (3.15%) is almost similar to the earlier observations of Bolka and Kumar *et al*. [16,17], who reported an EE content of 3.0-3.5%. Total ash content of *Azolla* obtained in this experiment was 21.67%. Ali and Leeson [23] reported a very high value of 36.12% ash in *Azolla*. However, other workers [6] recorded values almost similar to the present study. The NFE content of 32.25% recorded in this study was lower than the value reported by Parthasarathy *et al*. [24] as 38.85 to 44.06% NFE in *Azolla*. Parthasarathy *et al*. [24] also indicated that *Azolla microphylla* contained 2.11% calcium. Whereas, Ali and Leeson [23] found 0.31% phosphorus in *Azolla*. Both values are comparable to our findings. The variations in the nutrient composition of *Azolla* meal in different studies could be attributed to differences in the response of *Azolla* strains to environmental conditions such as temperature, light intensity, and soil nutrients which consequently affect their growth morphology and composition. Moreover, species difference of *Azolla* could alter their nutrient composition. Furthermore, contamination with epiphytic algae could also be important to such a degree as to affect the results of chemical composition.

**Table-2:** Nutrient contents of experimental diet (g/kg as DM).

Attributes	Experimental diet								

	Starter				Finisher				*Azolla*
	
	C	T1	T2	T3	C	T1	T2	T3	
DM	886.5	891.0	873.8	881.8	864.5	881.9	883.4	891.4	98.8
ASH	151.8	173.5	179.4	191.1	153.6	169.2	183.0	190.5	216.7
CP	231.6	233.4	237.9	240.0	196.8	199.9	202.2	206.9	256.4
EE	43.2	33.3	38.7	32.9	56.2	44.3	44.9	43.8	31.5
CF	40.1	40.9	41.2	43.0	43.9	44.2	44.9	45.8	172.9
NFE	533.3	518.9	502.8	493.0	549.5	542.4	525.2	508.5	322.5
NDF	303.0	311.9	335.0	310.0	356.7	339.9	330.1	345.1	511.5
ADF	112.7	92.2	100.7	109.3	122.2	114.5	119.8	129.1	433.8
Ca	13.8	16.0	16.9	17.2	10.2	13.4	14.0	14.6	111.2
P	9.7	10.5	10.9	11.4	9.0	9.8	10.2	10.4	59.1

DM=Dry matter, CP=Crude protein, EE=Ether extract, CF=Crude fiber, NFE=Nitrogen free extract, ADF=Acid detergent fiber, NDF=Neutral detergent fiber

### Blood biochemical parameters

All blood biochemical *viz*. glucose, creatinine, cholesterol, total protein, albumin, uric acid and triglycerides were found similar to control (Table-3) and within the normal reported values for broiler chicken [25]. The results showed that the treatment did not affect the plasma glucose concentration as the obtained values being in normal range [26]. Furthermore, indicated that carbohydrate metabolism was not affected by the diet [27]. It is known that triglycerides are important energetic products particularly used by chicks for growth performance [28,29]. But our study reflected no change in triglyceride content of blood. Total plasma proteins are a common parameter utilized to estimate the avian body condition. Moreover, albumin, one of the main serum proteins, serves as the most favorable source of amino acids for synthesis of tissue proteins [30]. Similar total protein and albumin content in the blood of all the treatment groups in our study represented normal body condition of all the experimental groups. Creatinine is another important indicator of protein metabolism, a by-product of phosphocreatine breakdown in skeletal muscle. Its concentration is directly proportional to muscle mass, related to age, physical activity and like the majority of blood chemistry constituents, is influenced by diet [31]. In our study, the plasma levels of albumin and creatinine were not affected by treatment and was found within physiological normal values [32].

**Table-3:** Blood biochemical parameters of Chabro birds at 8 weeks of age fed with different levels of *Azolla* meal.

Treatment	Glucose (mg/dl)	Creatinine (mg/dl)	Cholesterol (mg/dl)	Total protein (g/dl)	Albumin (g/dl)	Uric acid (mg/dl)	Tryglycerides (mg/dl)
T_1_	265.75	0.32	131.00	3.42	1.71	4.51	55.25
T_2_	261.25	0.34	131.75	3.40	1.73	4.64	56.75
T_3_	270.00	0.33	134.00	3.68	1.73	4.66	56.75
T_4_	265.00	0.34	135.00	3.52	1.67	4.47	56.25
SEM	4.02	0.02	3.66	0.10	0.02	0.08	1.20
p value	0.24	0.56	0.61	0.22	0.25	0.28	0.79

SEM=Standard error of mean

### Hematological parameter

All the hematological values were found similar between treatment groups except heterophil and lymphocyte which were found higher in T_2_ and T_3_ groups and eosinophil was found higher in T_3_ group than control (Table-4). The values of hematological indices obtained in this study were within the normal ranges reported by Anon [25] indicating that the birds were adequately nourished and thus not anemic or showing any sign of disease infection or parasitic problems. The heretophil: Lymphocyte (H:L) ratio quantifies the balance between the non-specific, fast acting defenses of heterophils and the antigen specific, slower-acting defenses of lymphocytes [33]. Therefore, H:L ratio is considered as a sensitive hematological indicator of stress response among chickens’ populations [34] and as a general biomarker relevant to immune function [33] in poultry. In the present study, similar H:L ratio in control and *Azolla* fed birds represented similar immune response and no stress due to *Azolla* supplementation.

**Table-4:** Hematological values of Chabro birds at 8 weeks of age fed with different levels of *Azolla* meal.

Treatment	Hb (g/dl)	PCV (%)	TLC (count/μL)×10^3^	DLC %				H:L

				Heterophil	Eosinophil	Lymphocyte	Monocyte	
T_1_	11.13	34.71	18.17	24.66^a^	1.93^a^	55.38^a^	6.89	0.44
T_2_	11.75	37.36	20.69	29.72^b^	2.10^a^	65.02^b^	8.82	0.46
T_3_	12.00	34.67	21.26	29.75^b^	2.72^b^	63.86^b^	8.79	0.47
T_4_	11.50	34.89	19.37	27.21^ab^	1.88^a^	60.19^ab^	7.90	0.45
SEM	0.25	1.40	1.13	1.09	0.08	2.11	0.65	0.01
p value	0.13	0.49	0.26	0.02	<0.01	0.03	0.17	0.63

Means bearing different superscript in a column differ significantly (p<0.05). SEM=Standard error of mean, Hb=Hemoglobin, PCV=Packed cell volume, TLC=Total leukocytes count, DLC=Differential leukocytes count, H:L=Heretophil:lymphocyte

### Liver enzyme and macro mineral status

Liver enzymes and macro mineral content in blood were found similar in all the treatment groups except aspartate aminotransferase (AST) which was found higher in T4 group than control (Table-5). Enzyme activities in birds are variable and originate from different organs. In poultry, AST and alanine aminotransferase (ALT) are synthesized in muscle, skeletal and cardiac, and in the second order in the liver [35]. No significant differences between the dietary treatments were noticed for ALT enzymes (p>0.05), the plasma concentration of ALT was within physiological normal values [36,37]. For the differences in AST values, Armand [38] has asserted that fluctuations in the serum levels of AST values are usually very difficult to interpret because of the wide distribution of this enzyme in avian tissues. The AST values obtained in this study, however, fall within the range of 105-143.5 IU/L reported by Anon [25]. Minerals are essential for broiler growth, and they are involved in many digestive, physiological and biosynthetic processes within the body. In the present study, the plasma Ca, P and Mg concentration was in normal range [26,32,36] and not affected by the treatment (p>0.05).

**Table-5:** Liver enzyme and macro-minerals of blood of Chabro birds at 8 weeks of age fed with different levels of *Azolla* meal.

Treatment	AST (IU/L)	ALT (IU/L)	ALP (IU/L)	Ca (mg/dl)	P (mg/dl)	Mg (mg/dl)
T_1_	115.22^a^	10.33	261.00	8.28	6.43	1.75
T_2_	118.03^a^	10.23	257.25	8.68	6.53	2.00
T_3_	128.37^ab^	10.15	255.00	8.48	6.65	1.95
T_4_	137.23^b^	10.18	257.25	8.20	6.48	1.78
SEM	6.46	0.31	5.06	0.15	0.12	0.08
p value	0.02	0.98	0.86	0.15	0.58	0.09

Means bearing different superscript in a column differ significantly (p<0.05). SEM=Standard error of mean, ALT=Alanine aminotransferase, AST=Aspartate aminotransferase, ALP=Alkaline phosphatase

### Immunocompetence traits

The humoral immune responses were found similar in all experimental groups (Table-6). This finding may be supported by similar H:L ratio in control and *Azolla* fed birds representing similar immune response. The H:L ratio quantifies the balance between the non-specific, fast acting defenses of heterophils and the antigen specific, slower-acting defenses of lymphocytes [33] thus acting as an indicator of stress response among chickens’ populations [34] and as a general biomarker relevant to immune function [33] in poultry. In contrary, Prabinaand Kumar [39] reported higher antibody tire value against Ranikhet virus in birds that were administered with dried *Azolla* at 10% level in comparison to the birds which took dried *Azolla* at 7.5% level. Similarly, Dhumal *et al*. [40] reported feeding *Azolla* meal in broiler improved the antibody titer values as compared to control group at 35^th^ days of age in commercial broilers. Although antibody titer was found similar in all the experimental groups in the present study, cell-mediate immune response (response to PHA-P) was found higher in T_2_, T_3_ and T_4_ than control(p<0.05) (Table-6). Similar observation was reported by Bhattacharya *et al*. [41] who fed commercial broiler with 4.5% and 5.5% *Azolla* meal and found 5.5% replacement group showed higher FI than control.

**Table-6:** Humoraland cell-mediate immune response of Chabro birds at 6^th^ week of age fed with different levels of *Azolla* meal.

Treatment	HA	IgG	IgM	Foot web index
T_1_	7.33	2.83	4.50	0.50^a^
T_2_	7.50	3.50	4.00	0.65^b^
T_3_	8.00	3.33	4.66	0.86^c^
T_4_	7.50	3.00	4.50	0.68^b^
SEM	0.37	0.31	0.50	0.02
p value	0.62	0.43	0.80	<0.01

Means bearing different superscript in a column differ significantly (p<0.05). SEM=Standard error of mean, HA=Hemagglutination assay, Ig=Immunoglobulin

## Conclusion

It has been observed that *Azolla* meal can replace commercial broiler feed upto 7.5% level without causing any adverse effect in blood biochemical parameters and hematological parameters. Moreover, higher cell-mediated immune response in *Azolla* replacement group indicated immune-modulatory effect of *Azolla* meal. In future, *Azolla* meal may be used as unconventional feed ingredient in poultry feed to reduce feed cost, especially in backyard poultry farming.

## Authors’ Contributions

DBM planned and carried out research work for his MVSc thesis program in collaboration with guide DR and advisory members AB and MK. VK, RK and SV helped DBM in analyzing blood samples for hematological and biochemical parameters. All authors participated in draft and revision of the manuscript. All authors read and approved the final manuscript.
